# Sustained mood improvement with laughing gas exposure (SMILE): a randomised, placebo-controlled pilot trial of nitrous oxide for treatment-resistant depression

**DOI:** 10.1192/bjo.2025.10823

**Published:** 2025-09-12

**Authors:** Karim S. Ladha, Jiwon Lee, Gabriella F. Mattina, Janneth Pazmino-Canizares, Duminda N. Wijeysundera, Fatemeh Gholamali Nezhad, Vanessa K. Tassone, Fathima Adamsahib, Wendy Lou, Sidney Kennedy, Venkat Bhat

**Affiliations:** Department of Anesthesiology and Pain Medicine, University of Toronto, Toronto, Ontario, Canada; Department of Anesthesia and Pain Management, Women’s College Hospital, Toronto, Ontario, Canada; Department of Anesthesia and Pain Management, Toronto Western Hospital, Toronto, Ontario, Canada; Li Ka Shing Knowledge Institute, St Michael’s Hospital, Toronto, Ontario, Canada; Department of Anesthesia, St Michael’s Hospital, Toronto, Ontario, Canada; Temerty Faculty of Medicine, University of Toronto, Toronto, Ontario, Canada; Interventional Psychiatry Program, St Michael’s Hospital, Unity Health Toronto, Toronto, Ontario, Canada; Institute of Medical Science, University of Toronto, Toronto, Ontario, Canada; Biostatistics Division, Dalla Lana School of Public Health, University of Toronto, Toronto, Ontario, Canada; Department of Psychiatry, Temerty Faculty of Medicine, University of Toronto, Toronto, Ontario, Canada; Neuroscience Research Program, St Michael’s Hospital, Toronto, Ontario, Canada

**Keywords:** Treatment-resistant depression, nitrous oxide, randomised controlled trial, RCT, TRD

## Abstract

**Background:**

Nitrous oxide may possess antidepressant effects; however, limited data exist on repeated administrations and active placebo-controlled studies in treatment-resistant depression (TRD).

**Aims:**

We aimed to test the feasibility of a randomised controlled trial examining a 4-week course of nitrous oxide or midazolam, an active placebo.

**Method:**

In this randomised, active, placebo-controlled pilot trial, 40 participants with TRD were assigned either a 1-h inhalation of 50% nitrous oxide plus intravenous saline (*n* = 20) or a 1-h inhalation of 50% oxygen plus intravenous midazolam (0.02 mg/kg, up to 2 mg; *n* = 20) once weekly, for 4 weeks. Feasibility was assessed by examining rates of recruitment, withdrawal, adherence, missing data and adverse events. The main measure of clinical efficacy was the change in depression severity (Montgomery–Åsberg Depression Rating Scale (MADRS)) score from baseline to day 42.

**Results:**

The recruitment rate was 22.3% (95% CI 16.9–29.0). Withdrawal rates were 10% (95% CI 2.8–30.1) in both groups and adherence rates were 100.0% (95% CI 82.4–100) in the nitrous oxide group and 94.4% (95% CI 74.2–99.0) in the placebo group. There were no missing primary clinical outcome data in either group (0.0%, 95% CI 0.0–17.6). MADRS score changed by −20.5% (95% CI −39.6 to −1.3) in the nitrous oxide group and −9.0% (95% CI −22.6 to 4.6) in the placebo group. Nearly all adverse events were mild to moderate and transient.

**Conclusions:**

The findings support the feasibility and necessity of conducting a full-scale trial comparing nitrous oxide and midazolam in patients with TRD.

Treatment-resistant depression (TRD) represents a subset of major depressive disorder (MDD) that is characterised by an inadequate response to two trials of antidepressant treatments.^1^ Up to 30% of patients with MDD are treatment-resistant.^2,3^ The likelihood of achieving positive outcomes decreases with subsequent treatment trials.^4^ Compounding this issue, TRD poses significant economic and social burdens because of its high global prevalence, medical costs and associated disability, morbidity and mortality.^5–9^ Taken together, there remains a significant unmet need for novel treatments with different mechanisms of action that can more effectively address the symptoms of TRD.

N-methyl-D-aspartate (NMDA) receptors contribute to the pathophysiology of MDD and may represent promising treatment targets for MDD and TRD.^10,11^ Accordingly, esketamine, an NMDA receptor antagonist, is currently approved for the treatment of TRD by the US Food and Drug Administration.^12^ In addition to targeting novel mechanisms, ketamine holds the benefit of exerting rapid-onset antidepressant effects unlike existing antidepressants, which often take weeks to show clinical effects.^13,14^ However, ketamine is associated with psychotomimetic side-effects.^15^ Furthermore, a subset of patients with TRD show inadequate response to ketamine, highlighting the need for alternative treatments.^16^

Another clinically available NMDA receptor antagonist is nitrous oxide (‘laughing gas’), a safe, low-cost, inhalational anaesthetic with widespread clinical use.^17,18^ Nitrous oxide is one of the oldest medications still in clinical use today, and has an established safety profile when given for short periods and repeated administrations are separated by >3 days.^19^ Recent randomised controlled trials (RCTs) suggest that it may provide rapid relief of symptoms of TRD.^20–26^ Studies show that a single administration of nitrous oxide produces rapid-onset antidepressant effects as early as 2 h in patients with TRD, lasting up to 2 weeks.^20–26^ However, it remains unclear whether repeated administrations of nitrous oxide over time in TRD provide accrual of antidepressant effects, as suggested by a prior nitrous oxide study in MDD.^20^ Furthermore, the aforementioned studies on nitrous oxide in TRD had short follow-ups and employed inadequate blinding, using standard placebo treatments that potentially allowed participants to distinguish nitrous oxide from its comparator by its psychoactive effects.^20–23,25,26^ In summary, previous studies have been limited by small sample sizes, short duration of follow-ups, single dosing sessions and inadequate blinding.

Definitive evidence for the use of nitrous oxide in TRD requires a parallel-arm RCT comparing an adequate dose and duration of nitrous oxide against an active placebo. To help inform the design of this full-scale trial, we conducted a pilot study to evaluate the feasibility of an RCT examining a 4-week treatment involving once weekly administration of nitrous oxide compared with the active placebo, midazolam, with follow-up at 6 weeks after the first treatment. We also evaluated preliminary efficacy of the treatment. We hypothesised that nitrous oxide would be well-tolerated by participants and would alleviate depressive symptoms compared with placebo at the 6-week follow-up.

## Method

The authors assert that all procedures contributing to this work comply with the ethical standards of the relevant Canadian National and Institutional Committees on Human Experimentation and with the Helsinki Declaration of 1975, as revised in 2013. All procedures involving human patients were approved by the St. Michael’s Hospital Research Ethics Board (study #21-096), registered on ClinicalTrials.gov (identifier NCT04957368) and previously published in a study protocol paper.^27^ Reporting followed the Consolidated Standards of Reporting Trials (CONSORT) extension to randomised pilot and feasibility trials.^28^

### Study design

This was a double-blinded (patient and outcome assessor), randomised, active placebo-controlled pilot trial in which participants were randomly assigned in a 1:1 ratio to receive either nitrous oxide or the active placebo, midazolam.

### Participants

Forty patients with TRD, aged 18–65 years, were recruited from the Interventional Psychiatry Program at Unity Health Toronto – St. Michael’s Hospital in Toronto, Canada, between 15 October 2021 and 27 December 2023. The Interventional Psychiatry Program receives out-patient referrals from psychiatrists and family physicians from across the province of Ontario. Informed written consent was obtained from all participants. Inclusion criteria were as follows: (a) meeting DSM-5 criteria for MDD, (b) experiencing a current major depressive episode (MDE) as confirmed by the Mini-International Neuropsychiatric Interview (MINI) for DSM-5,^29^ (c) scoring >17 on the Hamilton Rating Scale for Depression (HRSD),^30^ (d) failure to respond to two trials of antidepressant therapy of adequate dose and duration during the current depressive episode, (e) abstinence or use of highly effective or double-barrier contraceptive methods in women of childbearing potential and (f) capacity to provide informed consent. Exclusion criteria were as follows: (a) acute suicidality (score ≥3 on HRSD item 3, (b) MDE in individuals with bipolar disorder, (c) current substance misuse or dependence and/or history of alcohol misuse or dependence within the past year, (d) dementia, (e) current or lifetime history of schizophrenia or schizoaffective disorder, (f) current history of dissociative disorders, (g) history of hypersensitivity or allergy to any ingredients in the study formulations, (h) contraindication to nitrous oxide or midazolam, (i) use of centrally acting medicinal products or central nervous system depressants, (j) pregnant or breastfeeding women, (k) electroconvulsive therapy or ketamine treatment within the current depressive episode, or (l) unwilling to maintain the current antidepressant regimen.

### Randomisation and blinding

An online random number generator was used to generate the allocation sequence in random permuted blocks of varying sizes (4–6) without stratification on baseline characteristics. The allocation sequence was accessible solely to the Research Pharmacy at St. Michael’s Hospital, which prepared the study package upon enrolment of each participant. Each study package comprised either midazolam or normal saline vials, placed in an opaque paper bag to maintain blinding of study staff. The clinician administering the study intervention was unblinded to ensure proper delivery of the assigned intervention (i.e. set appropriate gas flows) and for safety reasons in the case of an adverse event requiring immediate resuscitation. Participants, outcome assessors, data analysts and all other clinicians were blinded to allocation assignment.

### Interventions

Participants underwent a 4-week course of weekly administered active treatment or placebo. The active treatment comprised a 1-h inhalation of nitrous oxide at an inspiratory concentration of 50%, along with concurrent intravenous saline. The placebo comprised a 1-h inhalation of 50% oxygen combined with concurrent intravenous midazolam (0.02 mg/kg, up to 2 mg). The use of midazolam as an active placebo is supported by a prior nitrous oxide study in treatment-resistant bipolar depression and several ketamine studies in TRD, as midazolam has psychoactive effects but lacks antidepressant effects.^31,32^ Participants continued to receive routine psychiatric care and treatment throughout the study, as determined by the participant’s psychiatrist. The study team asked that when clinically appropriate that limited changes be made to the participant’s treatment regimen while the participant was enrolled.

### Outcomes and assessments

Feasibility was assessed based on rates of recruitment, withdrawal, adherence, missing data and the number of adverse events. No pre-specified thresholds for feasibility were set *a priori*. The primary exploratory clinical outcome was the change in Montgomery–Åsberg Depression Rating Scale (MADRS)^33^ from baseline to 6 weeks. This outcome will also serve as the primary measure in a future full-scale trial, justifying its use as the main clinical measure in this pilot study. The MADRS was designed with the specific purpose of being sensitive to changes caused by an intervention over time,^33^ is one of the most commonly used measures of depression severity in RCTs and has psychometric properties that are equal or superior to other measures^34^. Other clinical measures included remission rate (MADRS score <10),^35^ response rate (≥50% decrease in MADRS score from baseline),^36^ minimal clinically important difference (decrease in MADRS score ≥6 from baseline),^37^ the Quick Inventory of Depressive Symptomatology (QIDS)^38^ and the General Anxiety Disorder-7 (GAD-7)^39^ and adverse events as assessed by the Toronto Side Effects Scale (TSES).^40^ Participants completed a baseline assessment, with follow-up assessments on days 1, 7, 14, 21, 28 and 42. A follow-up duration of 6 weeks was chosen to determine the if there were sustained benefits beyond the 2-week benefit described in previous studies.^41^ All diagnostic and rating scale assessments were performed by trained raters. Follow-ups were conducted by telephone interview, except in cases where data could be collected before intervention administration.

### Statistical analyses

Feasibility outcomes were summarised by using counts, rates and percentages. To summarise recruitment, the recruitment rate was calculated as the number of participants randomised divided by the number of participants assessed for eligibility as well as the number of patients recruited over time. The withdrawal rate was defined as the number of participants who withdrew divided by the number of participants who were randomised. The rate of adherence was calculated as the proportion of participants (excluding those that withdrew) who completed the full course of intervention. The proportion of missing data for the primary clinical outcome was calculated across participants who did not withdraw.

Clinical outcomes were analysed with the intention-to-treat principle. Descriptive statistics were used, including means and standard deviations for continuous variables, and counts and percentages for categorical variables. Two-sided 95% confidence intervals were calculated for proportions and percentages by using Wilson’s method. Between-group differences for continuous outcomes were assessed with analysis of covariance adjusted only for baseline scores. Statistical analyses were conducted with Stata version 17.0 (StataCorp, College Station, Texas, USA; available at https://stata.com). Given the pilot nature of the study, the sample size of 40 participants was selected based on logistical considerations to provide sufficient information on feasibility and effect size to inform the design of a future definitive trial. No formal sample size calculation was performed.

## Results

### Feasibility

**Fig. 1:**
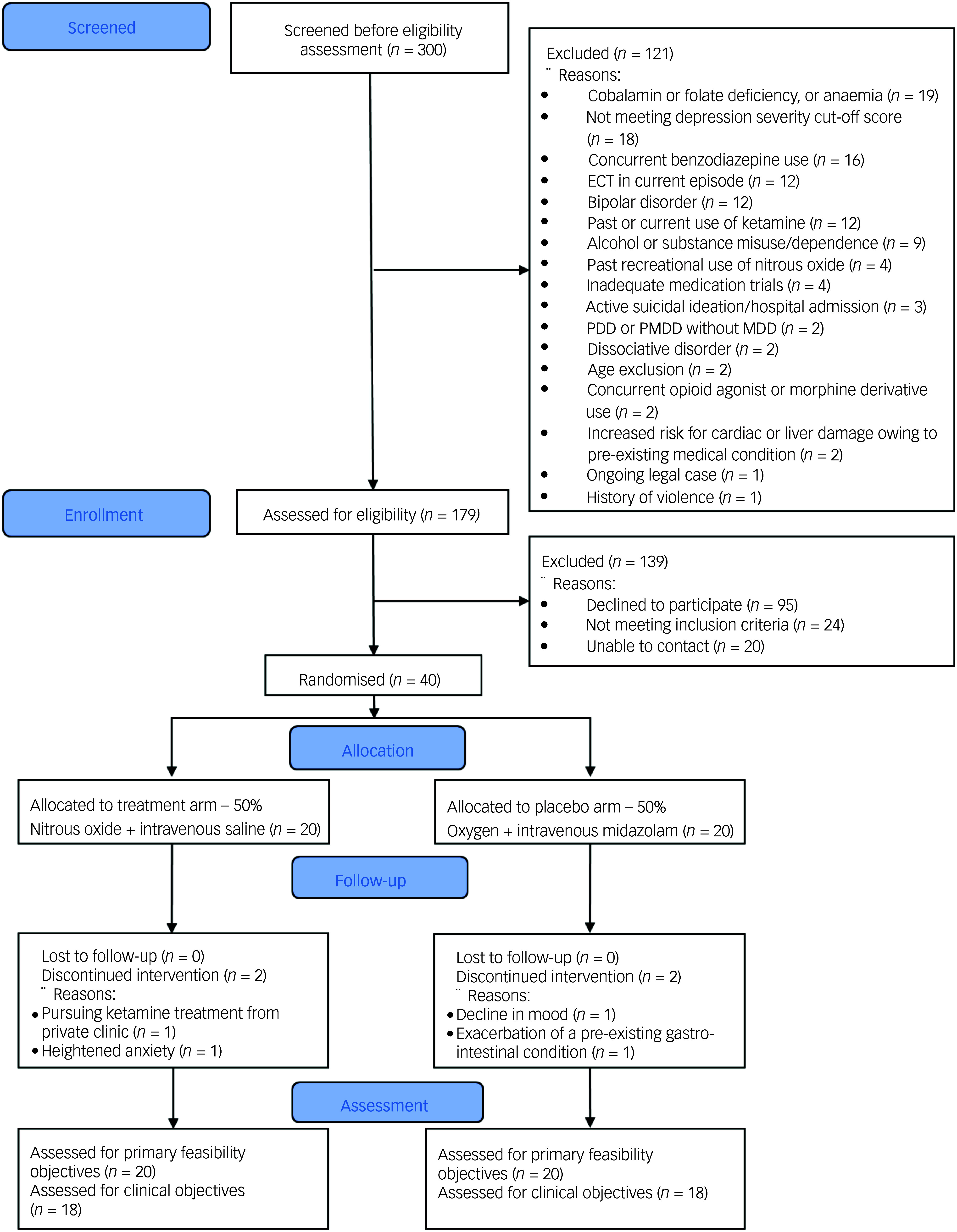
CONSORT flow diagram. ECT,electroconvulsive therapy; MDD, major depressive disorder; PDD, persistant depressive disorder; PMDD, premenstrual dysphoric disorder.

A total of 300 patients were initially screened, and 40 eligible participants were randomised to either the nitrous oxide group (*n* = 20) or placebo group (*n* = 20). The CONSORT participant flowchart is presented in Fig. 1 and the baseline demographics and clinical characteristics of the cohort are outlined in Table 1. Of the 179 patients assessed for eligibility, 22.3% (95% CI 16.9–29.0) consented to participate, which translates to approximately 1.5 patients per month recruited. Two participants (10%, 95% CI 2.8–30.1) withdrew from the nitrous oxide group because of heightened anxiety (*n* = 1) and choosing to pursue ketamine treatment (*n* = 1). Two participants (10%, 95% CI 2.8–30.1) withdrew from the placebo group because they experienced a decline in mood (*n* = 1) and exacerbation of a pre-existing gastrointestinal condition (*n* = 1). The 18 participants in the nitrous oxide group who did not withdraw fully adhered to the trial protocol (100.0%, 95% CI 82.4–100.0), i.e. received all four intervention administrations. Of the 18 participants in the placebo group who did not withdraw, 17 participants (94.4%, 95% CI 74.2–99.0) demonstrated full adherence to the intervention. The basis for non-adherence was one participant contracting COVID-19 and hence missing a treatment visit. There were no missing data (0.0%, 95% CI 0.0–17.6) for the main clinical outcome (MADRS score at day 42) across all the participants who did not withdraw. Adverse events were more frequent in the nitrous oxide group than the placebo group. Among the adverse events likely to be related to the trial, the most common events in both groups were tiredness/drowsiness, nausea/vomiting, headache and dizziness/lightheadedness (Table 2). In the nitrous oxide group, anxiety/chest tightness was additionally frequent. One participant in the nitrous oxide group experienced a serious adverse event, where they were admitted to hospital because of worsening mood.

**Table 1:** Baseline demographic characteristics of randomised participants who completed the study

Characteristic	Nitrous oxide (*n* = 18)	Placebo (*n* = 18)
Age, years, mean (s.d.)	44.4 (10.2)	41.6 (13.3)
Sex, *n* (%)		
Male	7 (39)	7 (39)
Female	11 (61)	11 (61)
Gender, *n* (%)		
Man	7 (39)	8 (44)
Woman	11 (61)	9 (50)
Other	0	1 (6)
BMI, kg/m^2^ mean (s.d.)	27.8 (5.8)	30.3 (6.3)
MADRS, mean (s.d.)	28.5 (6.7)	27.2 (3.9)
QIDS, mean (s.d.)	17.3 (4.2)	18.2 (4.1)
GAD-7, mean (s.d.)	13.7 (4.5)	12.4 (5.7)
Psychiatric comorbidities, *n* (%)		
Panic disorder	11 (61)	9 (50)
General anxiety	9 (50)	12 (67)
Agoraphobia	5 (28)	5 (28)
Social anxiety	4 (22)	8 (44)
PTSD	4 (22)	2 (11)
Obsessive–compulsive disorder	1 (6)	2 (11)
Bulimia	0	2 (11)
Antidepressant medications at baseline, *n* (%)		
Atypical antidepressants	8 (44)	7 (39)
SSRI	4 (22)	3 (17)
SNRI	4 (22)	4 (22)
Stimulant	3 (17)	7 (39)
Atypical antipsychotics	4 (22)	4 (22)
Sedative/hypnotic	4 (22)	2 (11)
Benzodiazepine	3 (17)	2 (11)
Anticonvulsant	1 (6)	2 (11)
TCA	0	2 (11)
Lithium	1 (6)	0
None	3 (17)	1 (6)

Numbers are listed as means and standard deviation or counts and percentages. BMI, body mass index; MADRS, Montgomery–Åsberg Depression Rating Scale; QIDS, Quick Inventory of Depressive Symptomatology; GAD-7, General Anxiety Disorder-7; PTSD, post-traumatic stress disorder; SSRI, selective serotonin reuptake inhibitor; SNRI, serotonin-norepinephrine reuptake inhibitor; TCA, tricyclic antidepressant.

**Table 2:** Frequency of adverse events by treatment group

Adverse event	Nitrous oxide (*n* = 20), *n* (%)	Placebo (*n* = 20), *n* (%)
Tiredness/drowsiness	10 (50)	12 (60)
Nausea/vomiting	9 (45)	5 (25)
Headache	8 (40)	5 (25)
Dizziness/lightheadedness	5 (25)	3 (15)
Anxiety/chest tightness	5 (25)	1 (5)
Bruising/pain at intravenous site	4 (20)	2 (10)
Body chills	2 (10)	2 (10)
Disorientation/confusion	2 (10)	0
Numbness/paresthaesia	2 (10)	0
Watering eyes/tearing	2 (10)	0
Burning sensation	1 (5)	0
Dry mouth	1 (5)	0
Dysphoria	1 (5)	0
Hospital admission (due to worsening mood)	1 (5)	0
Low blood pressure	0	1 (5)
Neck stiffness	1 (5)	0
Paranoia	1 (5)	0
Restlessness	1 (5)	1 (5)
Ringing in ears	0	1 (5)
Shakiness	1 (5)	0
Skin rash	1 (5)	1 (5)
Stomach cramps	1 (5)	0
Vivid dreams	0	1 (5)

### Clinical outcomes

When comparing changes from baseline to the day 42 follow-up (Table 3), the percentage change in MADRS score was −20.5% (95% CI −39.6 to −1.3) in the nitrous oxide group and −9.0% (95% CI −22.6 to 4.6) in the placebo group. The between-group difference in MADRS score at day 42, adjusted for baseline, was 3.0 (95% CI −1.8 to 7.9) in favour of nitrous oxide. The percentage change in QIDS score was −20.2% (95% CI −39.4 to −1.0) in the nitrous oxide group and −14.7% (95% CI −31.1 to 1.8) in the placebo group, with an adjusted between-group difference of 1.1 (95% CI −1.8 to 4.0). The percentage change in GAD-7 score was −18.2% (95% CI −42.9 to 6.5) in the nitrous oxide group and −17.0% (95% CI −49.9 to 16.0) in the placebo group, with an adjusted difference of 0.03 (95% CI −3.2 to 3.3). The full scores and changes over the course of the study of the MADRS, QIDS and GAD-7 at each follow-up visit are shown in Supplementary Fig. 1 available at https://doi.org/10.1192/bjo.2025.10823.

**Table 3:** MADRS, QIDS and GAD-7 scores at baseline (day 0) and day 42

	Nitrous oxide (*n* = 18)			Placebo (*n* = 18)			Adjusted between group difference at day 42
	Day 0	Day 42	Percentage change	Day 0	Day 42	Percentage change	
MADRS	28.5 (6.7)	22.7 (9.2)	−20.5% (95% CI −39.6 to −1.3)	27.2 (3.9)	24.8 (6.6)	−9.0% (95% CI −22.6 to 4.6)	3.0 (95% CI −1.8 to 7.9)
QIDS	17.3 (4.2)	13.8 (5.5)	−20.2% (95% CI −39.4 to −1.0)	18.2 (4.1)	15.5 (4.7)	−14.7% (95% CI −31.1 to 1.8)	1.1 (95% CI −1.8 to 4.0)
GAD-7	13.7 (4.5)	11.2 (5.5)	−18.2% (95% CI −42.9 to 6.5)	12.4 (5.7)	10.3 (6.4)	−17.0% (95% CI −49.9 to 16.0)	0.03 (95% CI −3.2 to 3.3)

Numbers are listed as means and standard deviation or percentage changes and 95% confidence intervals. Between group difference calculated by analysis of covariance adjusting for measurement at day 0. MADRS, Montgomery–Åsberg Depression Rating Scale; QIDS, Quick Inventory of Depressive Symptomatology; GAD-7, General Anxiety Disorder-7.

**Fig. 2:**
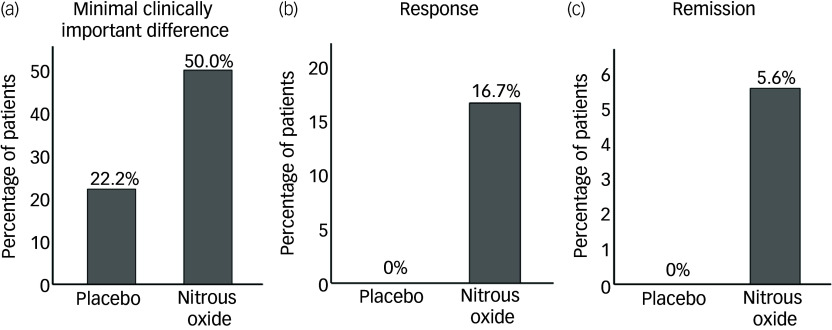
Percentage of patients achieving (a) a minimal clinically important difference (a decrease in MADRS score >6 from baseline), (b) a response (>50% decrease in the MADRS score from baseline) and (c) remission (MADRS score <10) by the day 42 follow-up in the placebo group (*n* = 18) and nitrous oxide group (*n* = 18). MADRS, Montgomery–Åsberg Depression Rating Scale.

At day 42 follow-up, 22.2% (4/18, 95% CI 9.0−45.2) of patients in the placebo group and 50.0% (9/18, 95% CI 29.0−71.0) of patients in the nitrous oxide group achieved a minimal clinically important difference of 6 points on the MADRS. The percentage of patients who achieved response as defined by a ≥50% decrease in MADRS score was 0.0% (0/18, 95% CI 0.0−17.6) in the placebo group and 16.7% (3/18, 95% CI 5.8−39.2) in the nitrous oxide group. Remission, defined as a MADRS score <10, was observed in 0.0% (0/18, 95% CI 0.0−17.6) of participants in the placebo group and 5.6% (1/18, 95% CI 10−25.8) of participants in the nitrous oxide group (Fig. 2).

## Discussion

This single-centre pilot study supports the feasibility of conducting an RCT investigating the efficacy of repeated administrations of nitrous oxide in TRD. Several criteria for feasibility were met. First, in terms of patient recruitment, we were able to consent approximately 1.5 patients per month, which would be sufficient for a future multi-site trial. Withdrawal rates and adherence rates were acceptable based on the limits of the 95% confidence intervals. Second, although adverse events were common in the nitrous oxide arm, they were generally mild in severity and transient. Third, differences in MADRS scores between the groups suggest that nitrous oxide may be beneficial for patients with TRD. Overall, these results support the feasibility of a multi-centre study to formally evaluate the use of repeated administrations of nitrous oxide in TRD.

The feasibility outcomes in this study were similar to other studies examining nitrous oxide. Given that our protocol required randomisation to an active placebo and four intervention administrations, there was concern about the acceptability and willingness of patients to participate in this study. Our results indicated that the recruitment rate was comparable to that of a previous crossover trial of nitrous oxide in TRD,^22^ and exceeded the recruitment rate in a similar 4-week, twice per week, study in MDD.^20^ Conversely, our recruitment rate was lower than that observed in a single nitrous oxide administration study^26^ and a crossover trial comparing nitrous oxide and oxygen.^23^ Withdrawal rates in both groups were comparable to, or lower than, those reported in prior nitrous oxide studies in TRD.^20–23,26^ Therefore, despite our protocol being more intensive than previous studies, this did not seem to substantially affect the ability to enrol participants.

The adverse events reported in the present study were consistent with those reported in previous nitrous oxide trials in MDD,^20,22,23,26^ as well as studies employing midazolam as a comparator to nitrous oxide in bipolar depression^31^ or to ketamine in TRD.^42–45^ The majority of the reported adverse events were mild or moderate in severity and transient in duration, underscoring the safety of the intervention. However, although prior nitrous oxide studies in MDD have not reported serious adverse events,^20,22,23,26^ one participant in this trial experienced a serious adverse event with the use of nitrous oxide, namely hospital admission for worsening mood, which was not temporally related to the intervention. This incident raises a potential safety concern for employing nitrous oxide as a treatment for symptoms of depression. As such, there is a need for further rigorous evaluation of the adverse events reported in this trial through future studies.

Caution should be taken in using outcome data in this feasibility trial to make inferences about potential treatment effects in a large multi-centre trial. Nonetheless, this pilot study adds to the accumulating literature on nitrous oxide as a treatment for TRD. Existing studies in TRD have primarily focused on assessing the efficacy of a single administration of nitrous oxide and have typically utilised a standard placebo comparator (i.e. oxygen),^20–23,25,26^ which may compromise blinding of participants.^23^ One study examined the effects of a 4-week, twice per week, treatment regimen with nitrous oxide; however, this study targeted patients with MDD rather than TRD specifically, and employed oxygen as a comparator.^20^ Compared with this, the present study achieved a higher recruitment rate despite several potential deterrents, including the longer follow-up period, the use of an active placebo arm and stricter inclusion criteria for patients with greater illness severity. Moreover, the very low withdrawal and high adherence rates despite the multi-week treatment course further supports the feasibility of this trial.

The lower bounds of the 95% confidence interval for the consent rate of patients assessed for eligibility (16.9%) was lower than targeted. Although we were still able to achieve a suitable recruitment rate, we intend to use this finding to identify aspects of the protocol to modify for the larger multi-centre trial. Specifically, our study team will engage with stakeholders to re-examine the need for specific exclusion criteria, including concurrent benzodiazepine use, electroconvulsive therapy in the current episode and past use of ketamine. Further, although reasons for declining participation were not formally captured, our study team noted that potential participants often had difficulty scheduling appointments, which prevented their participation. One reason for this is that there were limitations in terms of days when interventions could be administered, and thus we could not accommodate participant schedules. To mitigate these difficulties in a subsequent study, we will increase capacity to deliver the intervention, allowing for flexibility in scheduling, and intend to have the ability to arrange transportation for participants.

Several limitations and directions for future research should be noted. Although the sample size was sufficient for assessing feasibility, it does not allow for conclusions regarding efficacy of the interventions tested. Estimates generated in this study should be considered hypothesis-generating. This study was also conducted at a single centre and thus there may be unforeseen challenges in conducting the intervention at other centres. Therefore, the feasibility metrics observed may not be generalisable to a larger, multi-centre trial. Additionally, this study only had a 6-week follow-up (3 weeks following the last treatment session). It would be worthwhile in a subsequent, larger trial to extend follow-up beyond 3 weeks to evaluate the sustainability of antidepressant effects following a multi-week treatment with nitrous oxide. Although our study used an active comparator, unblinding of participants could have occurred because nitrous oxide potentially has a mild odour, and adverse events were more frequent in the nitrous oxide arm. Blinding was not formally assessed in this feasibility study and will be measured in the subsequent trial. Functional unblinding of participants may exaggerate the effect of nitrous oxide due to treatment expectations. Finally, the physician administering the intervention was not blinded to treatment allocation to ensure appropriate intervention delivery and due to safety considerations. This knowledge could have influenced their behaviour toward the participant and thus affected outcomes. At the same time, clinicians administering the intervention were not involved in outcome assessment to mitigate the effect of this potential bias.

In conclusion, this pilot study demonstrated the feasibility of an RCT examining a 4-week course of weekly administered nitrous oxide compared with an active placebo (midazolam). A future definitive trial is needed to assess the efficacy of repeated administrations of nitrous oxide versus midazolam for TRD.

## Supporting information

Ladha et al. supplementary material Ladha et al. supplementary material

## Data Availability

Requests for deidentified, aggregated data will be honoured whenever possible. Requests for anonymised individual participant data will be considered for researchers who provide a methodologically sound proposal for use of this information. Data will be available immediately following publication of this manuscript, with no anticipated end date. Analytic code will be made available, upon reasonable request.

## Supplementary material

The supplementary material is available online at https://doi.org/10.1192/bjo.2025.10823
