# Supplementary material for: Sustained mood improvement with laughing gas exposure (SMILE): a randomised, placebo-controlled pilot trial of nitrous oxide for treatment-resistant depression
Source: BJPsych Open. 2025 Sep 12;11(5):e208. doi: 10.1192/bjo.2025.10823 (PMC12451564; doi:10.1192/bjo.2025.10823)

**eFigure 1.** Scores on the clinical scales (A) MADRS, (B) QIDS, and (C) GAD-7 at each follow-up visit for the placebo group (n=18) and nitrous oxide group (n=18). MADRS=Montgomery–Åsberg Depression Rating Scale; QIDS=Quick Inventory of Depressive Symptomatology; GAD7=General Anxiety Disorder-7.


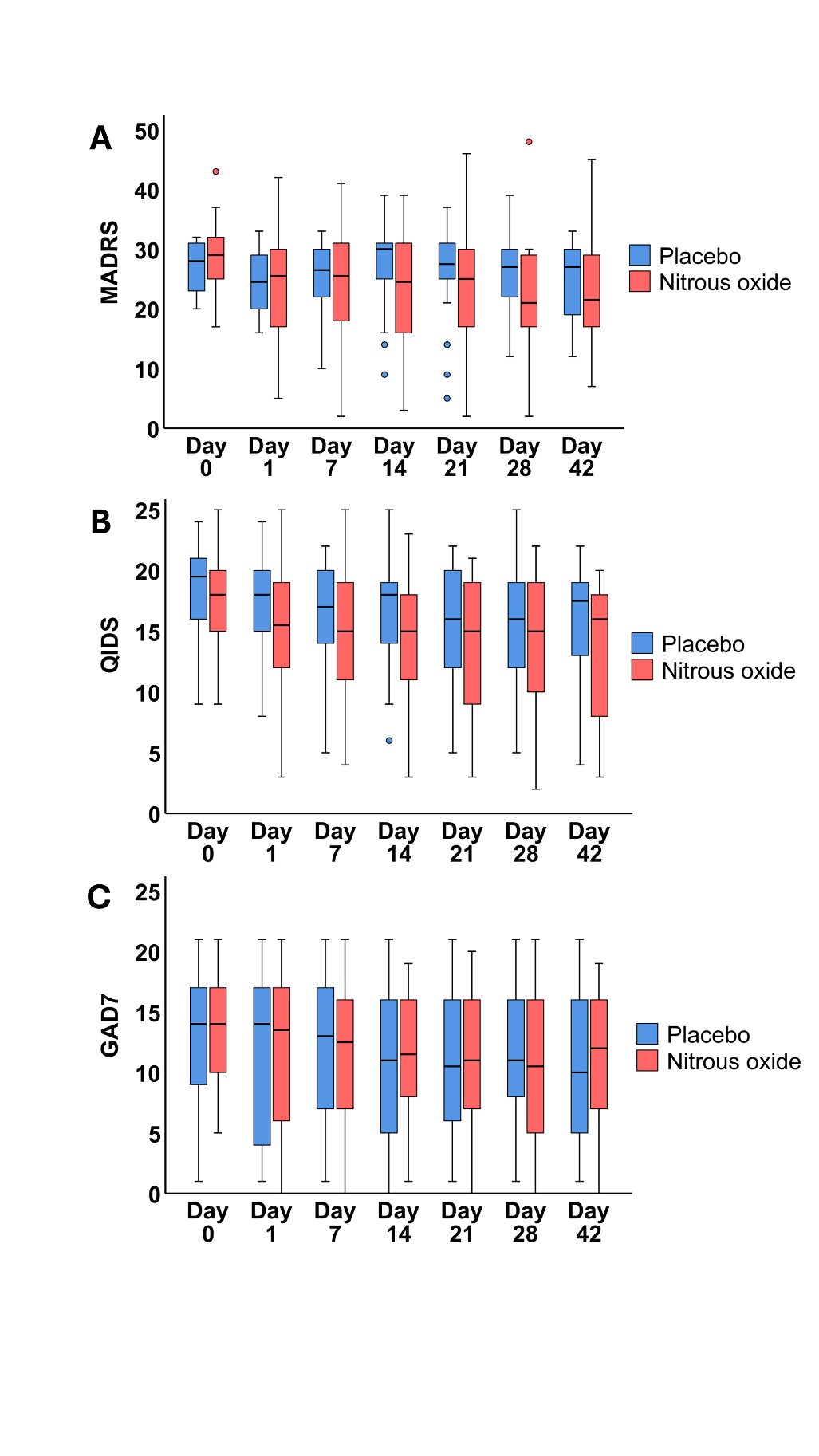

Supplement: Ladha et al. supplementary material [file S2056472425108235sup001.docx]
